# Proteomic Characterization of Changes in Mouse Brain Cortex Protein Expression at Different Post-Mortem Intervals: A Preliminary Study for Forensic Biomarker Identification

**DOI:** 10.3390/ijms25168736

**Published:** 2024-08-10

**Authors:** Martina Bonelli, Fabrizio Di Giuseppe, Nicola Tupone, Vimal Di Virgilio, Antonio Maria Catena, Marcello Locatelli, Giuliano Ascani, Gianluigi Giammaria, Renata Ciccarelli, Cristian D’Ovidio, Stefania Angelucci

**Affiliations:** 1 Department of Medicine and Aging Sciences, University “G. d’Annunzio” of Chieti-Pescara, Via dei Vestini 31, 66100 Chieti, Italy; martina.bonelli@unich.it (M.B.); cristian.dovidio@unich.it (C.D.); 2 Center for Advanced Studies and Technologies (CAST), University “G. d’Annunzio” of Chieti-Pescara, Via Luigi Polacchi 13, 66100 Chieti, Italy; fabrizio.digiuseppe@unich.it (F.D.G.); nicola.tupone@studenti.unich.it (N.T.); dvgiampi7@alice.it (V.D.V.); stefania.angelucci@unich.it (S.A.); 3 Department of Innovative Technologies in Medicine and Dentistry, University “G. d’Annunzio” of Chieti-Pescara, Via dei Vestini 31, 66100 Chieti, Italy; 4 Forensic Medicine, Facoltà di Medicina e Chirurgia Via Montpellier, Tor Vergata University, 100133 Roma, Italy; antonio.maria.catena@gmail.com; 5 Department of Pharmacy, University “G. d’Annunzio” of Chieti-Pescara, Via dei Vestini 31, 66100 Chieti, Italy; marcello.locatelli@unich.it; 6 UOSD Maxillofacial Surgery, Azienda Sanitaria Locale di Pescara, Via Renato Paolini 47, 65124 Pescara, Italy; giuliano.ascani@asl.pe.it; 7 Ravenna Medical Center (GVM Care and Research), 48124 Ravenna, Italy; dottgiammaria@gmail.com

**Keywords:** post-mortem interval (PMI), mouse brain cortex, proteins, forensic biomarkers, proteomics

## Abstract

Accuracy in the evaluation of death-induced tissue degradation for thanato-chronological purposes is strictly dependent on the condition of the biological source as well as on the precision of post-mortem interval (PMI) estimation. Thus, the optimization of tissue handling and identification of sensitive post-mortem biomarkers could help establish a timeline for post-mortem events. To this aim, we investigated the proteome changes in cortex samples of 6-week-old female SAMR1 mice over a post-mortem time course. After death, brain tissue was removed immediately (T0), and after 4, 8, 12, 24, and 32 h, four mice were used for each time period, and animals were maintained at 4 °C until brain removal. Dissected tissues were frozen at −80 °C until processed. Proteomic analysis, performed on samples related to early and late PMIs (<24 h and >24 h post-mortem, respectively) showed protein level changes as compared to T0 samples, with a remarkable increase in Calpain11 in the early PMI, as well as in Caspases 7 and 8 together with Gasdermin 3 in late PMI. These findings were confirmed by LIFT mass spectrometry technology and western blot analysis and, although requiring further investigation in other biological samples, suggest that these proteins could be considered as putative biomarkers of different PMIs.

## 1. Introduction

Following death, loss of homeostatic mechanisms leads to the decomposition of tissues with degradation of the constituent biomolecules, the identification and analysis of which can be useful to forensic pathologists to estimate post-mortem interval (PMI). Indeed, assessing a reliable time of death is an important task in daily forensic casework, as the success of the investigation very often depends on the establishment of a correct timeline of events [1]. However, to date, current methods are not able to determine a reliable and precise result, as those traditionally used differ depending on whether the analyses are performed within a short PMI (defined as early PMI, e-PMI) or a prolonged PMI (late PMI, l-PMI), the 24 h from the exitus representing the watershed between these two entities. Thus, for determining the e-PMI, it is common practice to evaluate consecutive abiotic cadaveric phenomena: temporal changes of hypostases, cadaveric rigidity, and body cooling, which, however, are influenced by intrinsic and extrinsic variables (i.e., environmental temperature, cause of death). Even for l-PMI evaluation, based on the study of transformative cadaveric phenomena, the latter is remarkably affected by variables both external and internal to the corpse. Moreover, in particular environmental conditions, special transformative phenomena can settle (mummification, saponification, corification, etc.), making dating PMI even more difficult.

The study of proteins in the forensic context by proteomic tissue analysis could overcome some of the shortcomings of the conventional techniques, as, unlike nucleic acids, proteins are molecules highly stable in well-defined physical and environmental conditions and can be preserved for prolonged periods. Proteomics also provides information on the physiological status of individuals, the presence of pathogens, and exposure to toxic substances. It also enables the identification of specimens of high biological value in the forensic context. Thus, all these characteristics make proteomics an emerging and powerful tool of great potential in forensic science [2]. Numerous studies have been conducted in the literature to try to identify the degradation pattern of proteins onto solid matrices, especially in relation to the investigation of animal skeletal muscle tissue, promoting a correlation between the qualitative and quantitative changes of proteins and the properties of meat, mainly for food purposes [3,4,5,6]. On the contrary, a few studies have been carried out that have the main objective of determining the correlation between proteomic data and PMI estimation, most of them being related to proteomic changes in peripheral tissues. Indeed, Gos and Raszeja investigated the activity of lactate and malate dehydrogenase in the human liver up to 35 days post-mortem, reporting a time-dependent decrease in them [7]. Some years later, Kikuchi et al. [8] showed an increase in the serum mobility group box-1 protein (HMGB1), expressed in various chronic inflammatory and autoimmune diseases, which exhibited high levels in cadavers stored at 4 °C. As well other studies confirmed the influence of late and early PMI on the levels of calmodulin-dependent kinase II (CaMKII), myristoylated alanine-rich C-kinase substrate (MARCKS), and calcineurin A (CnA) in lung [9] and muscle tissues [10]. Further studies focused on proteome tissue changes following different PMIs. Tavichakorntrakool et al., evaluating differences in the proteome of the human vastus lateralis muscle at 0, 2, 4, 6, 12, 24, and 48 h post-mortem and at 4 °C and 25 °C, confirmed that post-mortem proteome changes could be modified by temperature storage and individual characteristics [11].

Proteomic studies have also been performed on brain tissue, even though they are mainly used to investigate mechanisms and identify potential novel biomarkers and drug targets for neurological diseases [12]. Only a smaller number of investigations have been dedicated to cerebral post-mortem proteomic changes. Among these, Pla et al. measured the activities of rate-limiting glycolysis enzymes in post-mortem rat brains [13]. Many years later, a systematic study on post-mortem changes of rat brain proteome, carried out on tissue protein extracts from rats kept for several post-mortem times up to 72 h [14], highlighted the main alterations of structural proteins (i.e., neurofilament, dynamin-1, tubulin, and actin) as due to specific and/or nonspecific proteolytic mechanisms, strongly related to the proteome stability. Subsequently, the articles by two research groups [15,16] stand out, both being focused on human cerebrospinal fluid (CSF) as the biological source, where a lot of intracellular proteins showed variable expression levels in the pre- and post-mortem periods, suggesting a putative role of them as cell death biomarkers. Lastly, other studies [17,18,19], considering the pronounced biochemical changes occurring in the post-mortem period, were aimed at identifying some quality indicators markers (i.e., proteolytic activity, temperature, and time-dependent changes, etc.) in order to determine the stability of the proteome and therefore the integrity of a sample.

In the present work, a gel-based proteomic approach was used to profile changes in the brain cortex of mice examined under conditions that simulate the handling of autopsy material. The identification of the protein changes was performed at different post-mortem periods, distinguished as e-PMI (up to 12 post-mortem hours) and l-PMI (24–32 post-mortem hours). The primary aim was to identify potential new biomarkers useful for PMI assessment as well as additional parameters for sample quality measurements.

## 2. Results

### 2.1. Post-Mortem Mouse Brain Proteome Changes

**Figure 1:**
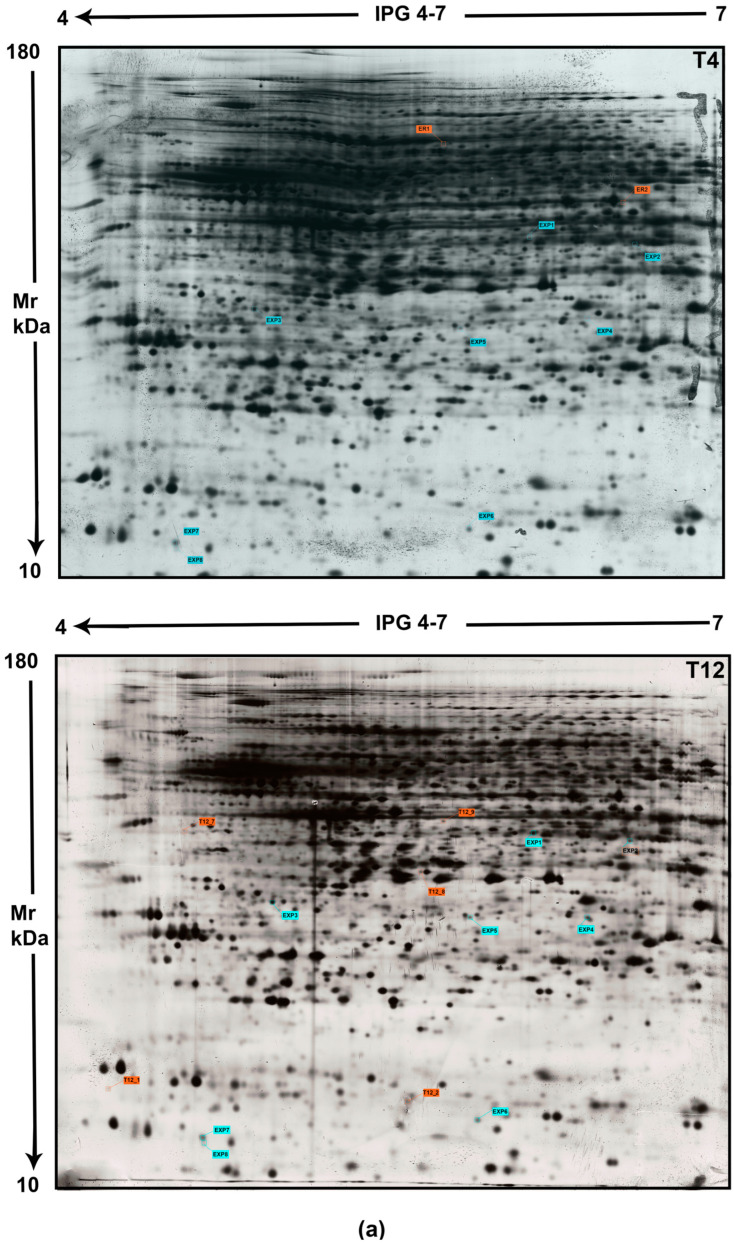

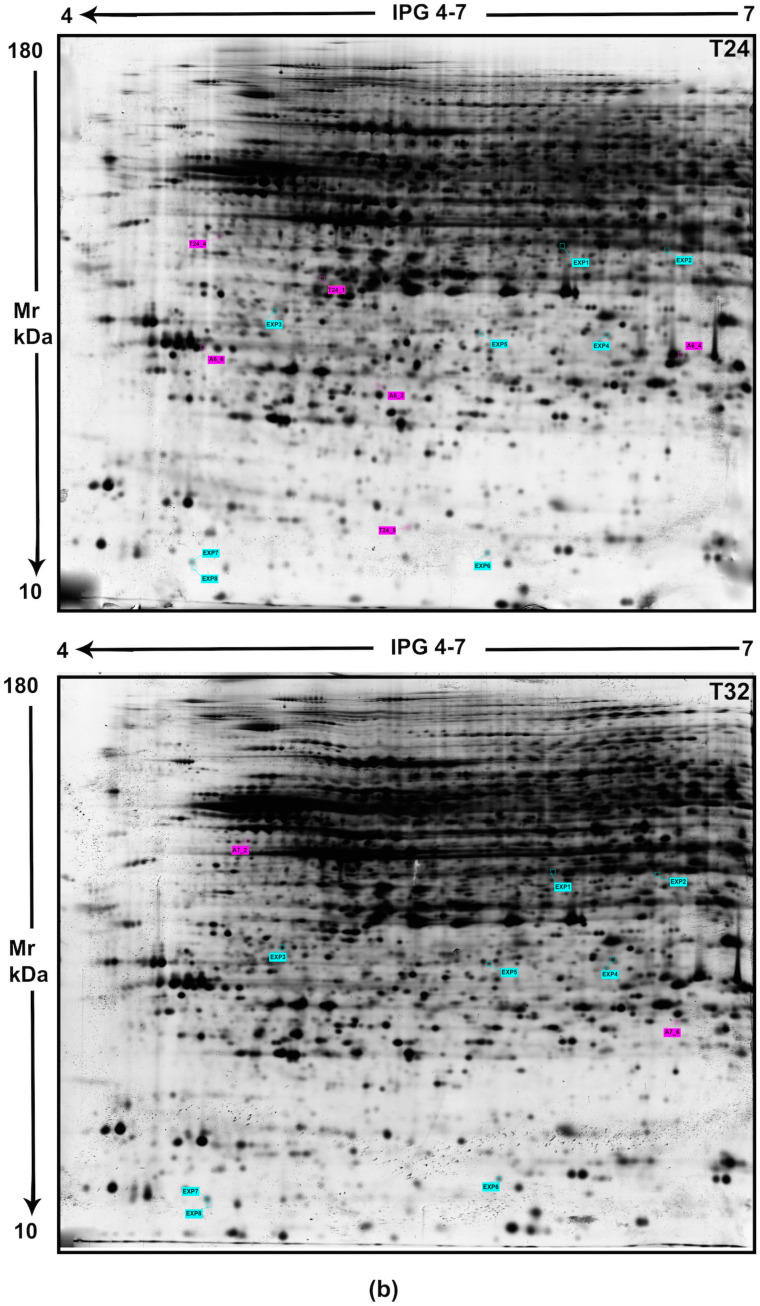
The representative proteome of mouse cortex for different PMIs. Cortical proteins were electrophoresed by immobilized pH 4–7 gradient (24 cm linear) on 12% homogeneous SDS-PAGE, then scanned by calibrated densitometer. All spots labeled on each gel are proteins chosen for mass spectrometry identification and reported in Table 1. In detail, T4 and T12 gels display protein spots expressed at e-PMIs (panel (**a**)), whereas in T24 and T32 gels, protein spots detected in l-PMIs are visualized (panel (**b**)). Spots listed in Table 1 as **EXP1,2,3,4,5,6,7,8** and marked on the gel with the label **cyan** represent common proteins detected in all 2D maps (T4, T12, T24, and T32), which were downregulated in mouse cortex over PMIs up to 32 h as compared to T0 map (Figure S1 in the Supplemental Materials). Unmatched spots in each gel are hallmarked in **orange** for the early stage (in Table 1 as **ER1,2,** **T12_1,2,7,8,9**) and **magenta** for the late stage (in Table 1 as **A6_3,4,6,** **A7_2,6, T24_1,4,5**).

Proteome analysis was carried out to identify post-mortem-induced changes in mouse brain cortex at different time points: 0, 4, 8, 12, 24, and 32 post-mortem hours indicated as T0, T4, T8, T12, T24, T32, respectively. The total protein content in each brain sample was determined. The values, reported as the mean ± S.E.M of protein concentrations obtained at each time point, were 5.8 ± 0.4 mg (T0), 4.3 ± 0.25 mg (T4), 6.2 ± 0.5 mg (T8), 5.2 ± 0.33 mg (T12), 6.2 ± 0.42 mg (T24) and 6.2 ± 0.4 mg (T32) of proteins. Aliquots (150 μg) of the total proteins were resolved by bidimensional electrophoresis (2DE), performing technical triplicates for each time point from T0 up to T32. Examples of these gels are reported in Figure 1 (see below) and Figure S1 (in the Supplemental Materials).

Each brain sample was individually analyzed on 2DE twice. All spots were distributed in a 4–7 pH range, molecular weight of separated proteins ranging from 10 to 180 kDa. Image analysis allowed to detect a total number of 2283 ± 12 spots at T0, 2487 ± 10 spots at T4, 2694 ± 14 spots at T8, 2987 ± 16 spots at T12, 3021 ± 20 spots at T24 and 3411 ± 8 spots at T32, respectively. These counts highlighted an increasing trend of the spot number over the post-mortem time course, as reported in Figure S2 (Supplemental Materials). Spot intensity was normalized by gel comparison, which measured the correlation coefficient. Each data point, when compared by normalized optical densities, showed a correlation coefficient of about 0.998.

### 2.2. Differences in the Mouse Cortex Protein Levels

**Figure 2:**
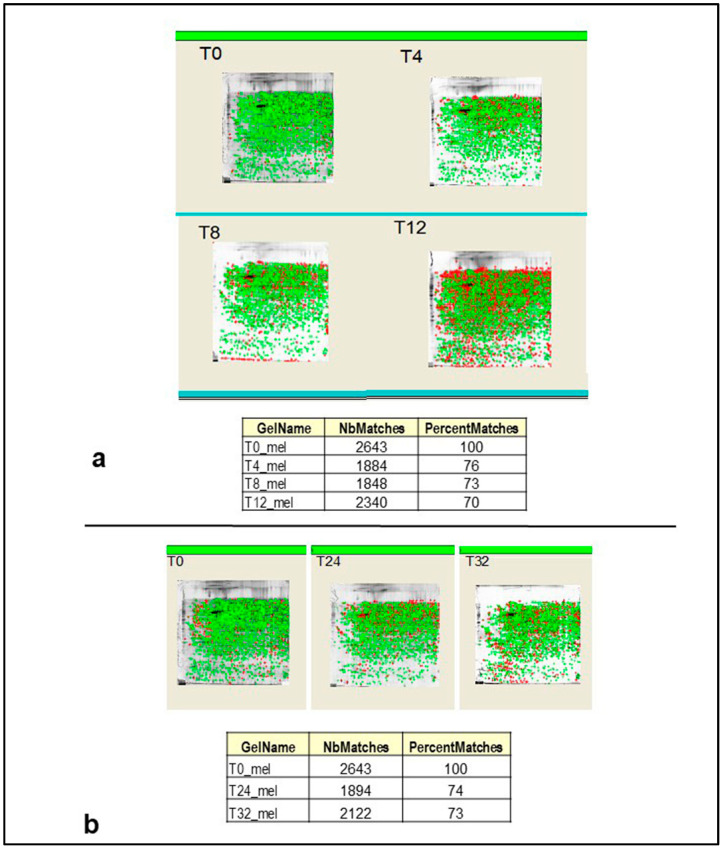
Statistical analysis of gel similarity. T0 2D gels were compared with those obtained at different time points. This comparison allowed us to identify a great number of matching spots, as reported in the table below, of gels related to e-PMI (**a**) and l-PMI (**b**). Matched protein spots are marked in green, whereas red spots represent unmatched proteins.

The cortical proteome was analyzed by comparing the spot intensities between 2D maps obtained at T0 and different PMIs. The statistical analysis of data, well known as % matching, showed high similarity among the spot intensities measured in samples collected at 4, 8, and 12 post-mortem hours as well as among those collected at 24 and 32 post-mortem hours (see Figure 2a,b), we grouped the most relevant changes in protein spot intensities over e-PMI (4–12 h) and l-PMI (24–32 h).

The gel comparison between the early stages’ and T0 2D maps also revealed an increasing number of unmatched spots. In detail, they were 13 in T4 vs. T0, 25 in T8 vs. T0, and 35 in T12 vs. T0. Likewise, there were 71 unmatched spots in T24 vs. T0 and 32 in T32 vs. T0. Among the unmatched spots, only those with a statistically significant intensity value (*p* < 0.05) and expression level ≥ 2 were selected for the subsequent identification in mass spectrometry (MS). In this way, 45 ± 3 and 51 ± 2 proteins spots were picked from early and late stages groups, respectively. Differentially expressed proteins were identified by MALDI-TOF/TOF MS analysis. A total number of 22 protein spots were unequivocally assigned. Table 1 lists these proteins distributed into three main groups according to their expression level over the post-mortem periods.

#### 2.2.1. Top Proteins Differentially Expressed in Mouse Cortex from 0 to 32 Post-Mortem Hours

In Table 1, group “a” includes seven proteins showing a decreased expression level in all examined PMIs. Therefore, they can be considered constitutive proteins, the expression of which was negatively modulated over the investigated PMIs.

This group includes two different isoforms of 26S proteasome regulatory subunits, namely isoforms 4 (PRS4) and 10B (PRS10). Both of them participate in various cellular processes, including cell cycle progression or DNA damage repair, and are involved in the maintenance of protein homeostasis by removing misfolded or damaged proteins. Another downregulated protein was choline transporter-like protein 3 (CTL3), which belongs to the solute carrier 22 (SLC22) family that includes six members deputed to transport human organic cations. It is expressed in human and rodent tissues (reviewed in [20]). Noteworthy, there was also a decreasing expression of 5-azacytidine-induced protein 2 (AZI2) connected with the antyzime family, involved in polyamine homeostasis through the ornithine decarboxylase (ODC) pathway [21]. As well, phosducin-like protein 2 (PDCL2) was downregulated in all analyzed PMIs. It is normally expressed at very low levels in the central nervous system of mammals and is functionally linked to the antinociception process [22]. Differently, the astrocytic phosphoprotein PEA-15 (PEA15) is expressed in all mammalian cell types and tissues. It is involved in the regulation of both cell proliferation and apoptosis through interaction with different pathways [23]. PEA15 was also found to be expressed in plaque-associated reactive astrocytes in post-mortem human Alzheimer’s disease (AD) tissue, so it was proposed as a biomarker of AD, along with other pathological hallmarks, to assess the severity of the disease progress at the time of death [24]. The last protein listed in the group “a” is a GA-binding protein subunit beta-1 (GAPBPB-1), a transcription factor that acts as a regulator of nuclear-encoded mitochondrial genes and the transcription process.

#### 2.2.2. Top Proteins Differentially Expressed in Mouse Cortex at Early PMI (from 0 to 12 Post-Mortem Hours)

Group “b” of Table 1 includes proteins with two different expression times over the early PMI. The first two proteins, namely phosphoglucomutase-2 (PGM2) and ATPase PAAT protein associated with ABC transporters (PAAT), were exclusively detectable from T0 up to T12, while the other five proteins were triggered from 12 post-mortem hours onwards, remaining at increased level up to 32 post-mortem hours. PGM2, a glycolytic enzyme, promotes the conversion of the nucleoside breakdown products ribose-1-phosphate and deoxyribose-1-phosphate to the corresponding 5-phosphopentoses. In addition, it plays a key role in the sub-pathway of 2-deoxy-D-ribose 1-phosphate degradation, contributing to the release of degradation end products such as hypoxanthine, xanthine, uracil, and uridine, which reached a maximum level after 24 post-mortem hours in the human brain, as described in the literature [25]. PAAT interacts with the three known mitochondrial inner membrane ABC proteins, ABCB7, ABCB8, and ABCB10, and functionally regulates the transport of ferric nutrients and heme biosynthesis. Significantly, PAAT deficiency promotes cell death, reduces mitochondrial potential, and sensitizes mitochondria to oxidative stress-induced DNA damage [26].

Two cytoskeletal proteins were upregulated in the early stage as well as in the late stage: vimentin-type intermediate filament-associated coiled-coil protein (VMAC) and actin cytoplasmic 1 (ACTB). VMAC is a type III intermediate filament (IF) protein assembled into cytoplasmic homo-polymeric and hetero-polymeric filaments with other type III and some type IV IFs. It is also considered as an immature astrocyte marker [27]. ACTB is a highly conserved protein that polymerizes to produce filaments that form cross-linked networks in the cytoplasm of cells.

Of the other three proteins with prolonged upregulation, the nucleotide exchange factor SIL1 precursor (SIL1) is an endoplasmic reticulum (ER)-resident 54 kD protein composed of 461 amino acids. SIL1 acts as the nucleotide exchange factor of ER chaperone protein Bip, which is a member of the heat shock protein 70 family and plays important roles in mediating the folding and assembly of nascent proteins, as well as the degradation of misfolded proteins [28]. Developmental pluripotency-associated protein 5A (DPA5A) is involved in the maintenance of embryonic stem (ES) cell pluripotency, although it is dispensable for self-renewal of pluripotent ES cells and the establishment of germ cells. Lastly, Calpain-11 (CAN11) is a calcium-regulated non-lysosomal thiol-protease, which catalyzes limited proteolysis of substrates involved in cytoskeletal remodeling and signal transduction. It is influenced by mitochondrial and other organelles’ (i.e., lysosomes and the ER) activity, which have an important function in the release and activation of death factors such as cathepsins, other calpain isoforms, and proteases [29].

#### 2.2.3. Top Proteins Differentially Expressed in Mouse Cortex at Late PMI (from 24 to 32 Post-Mortem Hours)

In addition to the five proteins reported in group “b” of Table 1, whose expression started in the e-PMI remaining elevated along the l-PMI, seven proteins were univocally upregulated at 24 h and 32 h in the l-PMI stage, even though we reported gasdermin-3 two times, as we found two isoforms for it.

Thus, over this period, a mitochondrial precursor of protein arginine methyltransferase (NDUFAF7) was detected, which is involved in the assembly and stability of mitochondrial NADH:ubiquinone oxidoreductase complex (complex I). The literature confirmed that it also triggers apoptosis in the initial phase and induces impaired clearance of apoptotic materials by inappropriate DNA methylation [30]. Another protein induced at 24 h post-mortem is nucleophosmin (NPM), which is involved in various cellular processes such as ribosome biogenesis, centrosome duplication, protein chaperoning, histone assembly, cell proliferation, and regulation of tumor suppressors p53/TP53 and ARF. It plays a key role in protein biosynthesis, exerting control over APEX1 endonuclease activity within nucleoli. This protein also negatively regulates the activation of EIF2AK2/PKR and suppresses apoptosis through inhibition of EIF2AK2/PKR autophosphorylation, being overexpressed in many tumors. Interestingly, NPM expression is elevated in the brain, where it is implicated in the regulation of neuronal viability. However, NPM overexpression in post-mitotic neurons can lead to cell degeneration/death [31].

Other proteins found after 24 h from death were the transcription elongation factor A protein-like 8 (TCAL8), a nuclear protein with a low human brain specificity, which may be involved in transcriptional regulation, and heparan sulfate glucosamine 3-*O*-sulphotransferase 6 (HS3S6), an enzyme predominantly expressed in neurons, wherein it generates rare 3-*O*-sulphated domains of unknown functions.

Among the remaining proteins of note was the high expression level of gasdermin-A3 (GSDA3), a pore-forming protein that causes membrane permeabilization and pyroptosis and is therefore considered a putative necrosis biomarker [32]. GASDA3 can be expressed in multiple forms with different pI ranges (from 5.53 to 5.75); in fact, as stated above, we found two GSDA3 isoforms listed in Table 1, group “c.” Also, it is of note the presence of two isoforms of caspases, the isoforms 7 (CASP7) and 8 (CASP8), that play a key role in programmed cell death by acting as a molecular switch for apoptosis, necroptosis, and pyroptosis [33].

### 2.3. Protein Sequence Validation by LIFT Technology and Western Blot Analysis of Some Peculiar Proteins Selected among the Others in the e-PMI and l-PMI

We further analyzed some of the identified proteins reported in Table 1. By using the LIFT technology (as described in the Section 4), it was possible to obtain ion parental masses from PMF spectra and ascertain univocal peptide sequences (reported in red) for each selected protein, which are shown in Table 2.

**Figure 3:**
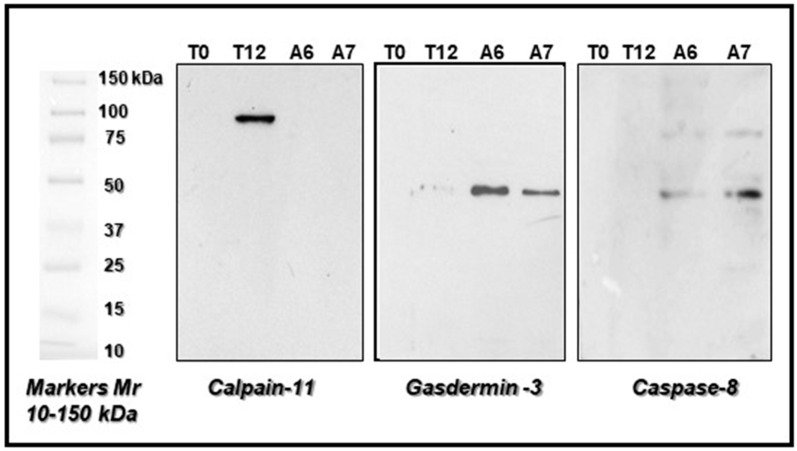
Representative western blots showing the expression of proteins specifically induced at early PMI (from 0 to 24 h= T12) like Calpain-11 and at late PMI (from 24 to 32 h = A6, A7) such as Gasdermin 3 and Caspase 8, previously identified by MS and reported in Table 1 together with other proteins. Protein lysates (30 µg) were loaded for each electrophoretic lane.

Furthermore, western blot analysis using specific antibodies against the selected three proteins mentioned above confirmed the identification of these proteins obtained by mass spectrometry techniques (Figure 3). Therefore, by these two methods, we are sure that the indicated proteins had an intact amino acid sequence and could be used as putative biomarkers for the different PMIs taken into consideration.

### 2.4. Bioinformatic Analysis of Mouse Brain Proteins from Early to Late PMI

**Figure 4:**
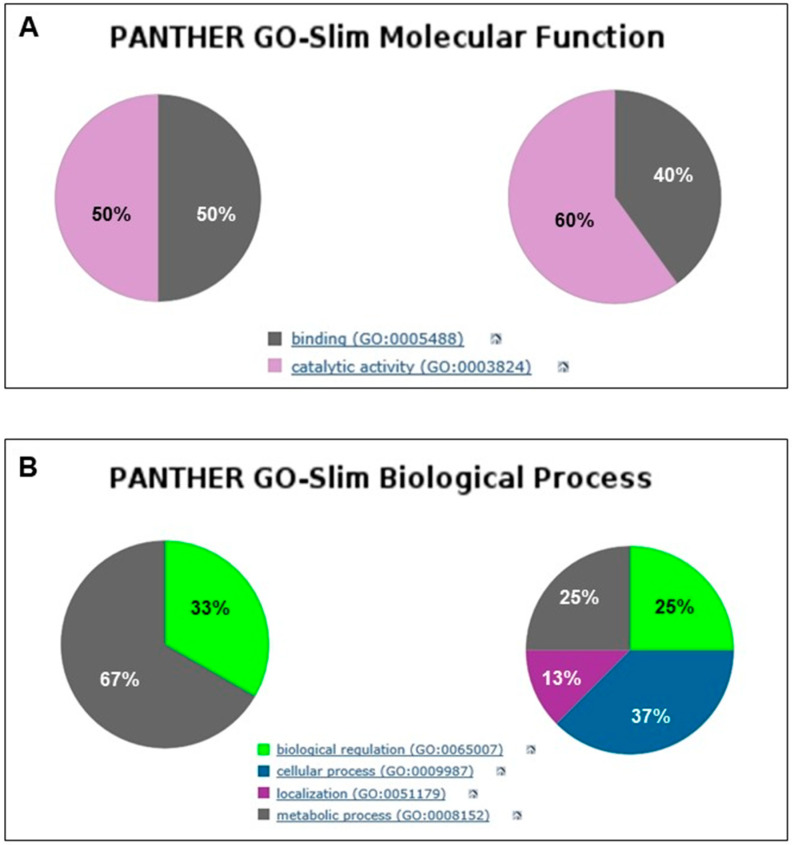

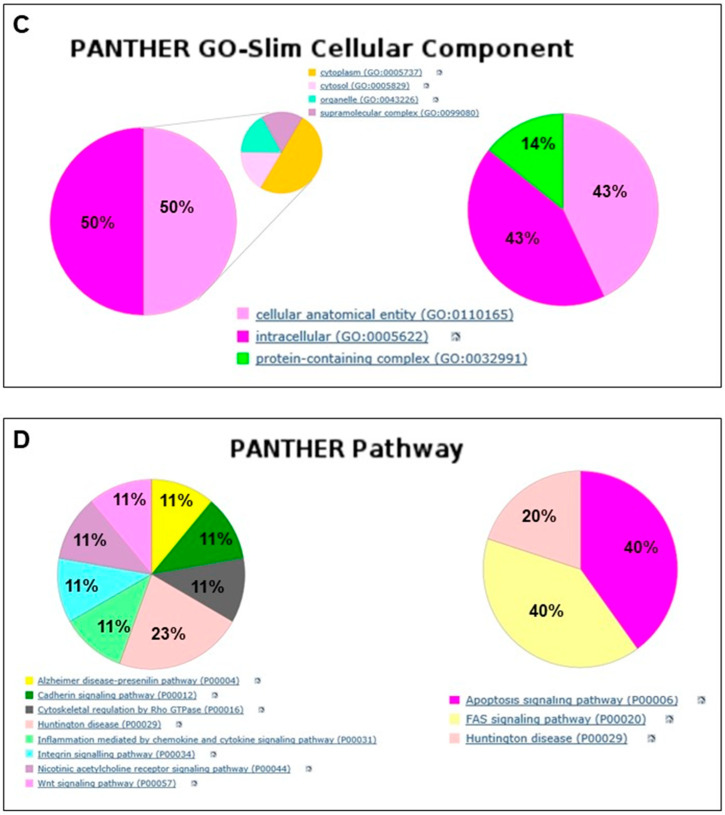
We launched the proteins identified by MS at early and late PMIs in the Gene Ontology database. Using Panther classification, we obtained the pies reported in panels (**A**–**D**), which show the percentage of cortical protein distribution between early and late PMIs based on molecular function (**A**), involvement in biological processes (**B**), cellular localization (**C**) and molecular pathway (**D**).

Gene Ontology (GO) analysis of all mouse cortex proteins newly expressed over the e-PMI (PGM2 and PAAT) and l-PMI (VMAC, DPA5, SIL1, CAN11, ACTB) provided a general overview of their molecular activity. There was an equal distribution of them in two main categories showing binding (50%) and catalytic activity (50%) in e-PMI (Figure 4A), while the proteins in the l-PMI stage (NDUFAF7, NPM, GSDA3, CASP8, HS3S6, CASP7, GSDA3 and TCAL8) showed a greater % distribution among those with catalytic activity (Figure 4A).

As for the role in biological processes, some proteins identified in the e-PMI exhibited a prevalent involvement in metabolic processes (67%), while others would participate in biological processes to an extent similar to that of some proteins identified in l-PMI. Most of the latter, however, were also involved in further biological processes (Figure 4B).

In terms of cellular localization, proteins of e-PMI are equally distributed between intracellular and cellular anatomical entities (Figure 4C); in particular, DPA5A and CAN11 represent the predominant cytoplasmic proteins. Noteworthy is the presence of l-PMI in the pie chart of a new category: protein-containing complex (14%), represented by NPM.

Finally, according to molecular pathways, in the e-PMI stage, various categories are identified: Alzheimer’s disease pathway, cadherin signaling pathway, cytoskeletal regulation by Rho GTPase, inflammation mediated by chemokine and cytokine signaling pathway, integrin signaling pathway, nicotinic acetylcholine receptor signaling pathway, Wnt signaling pathway, and Huntington disease pathway. Only the latter is still present in the l-PMI stage together with the other two categories, apoptosis signaling pathway and FAS signaling pathway (Figure 4D).

### 2.5. Functional Analysis of Proteins Expressed in the Early and Late PMIs

**Figure 5:**
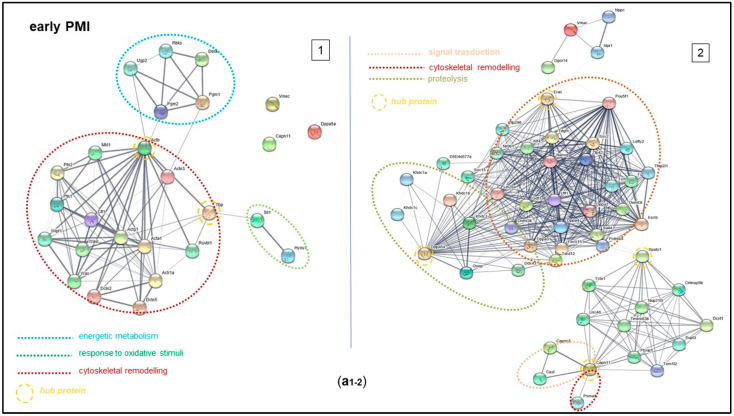

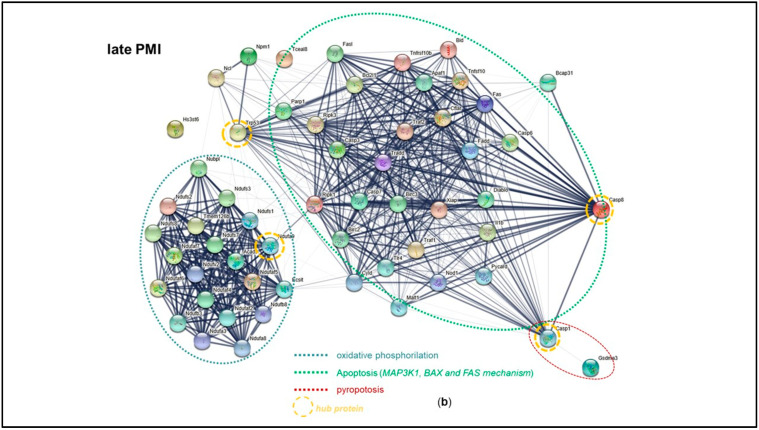
PMI Protein Interaction Network at early (**a1**,**a2**) and late PMIs (**b**). Using STRING (http://string-db.org), the functional links of the proteins expressed in mouse cortical samples were constructed. Proteins shown as spheres and labeled with gene names represent the nodes, whereas nodes associated with each other are linked by edges representing their interaction. Thicker lines indicate stronger associations. Main clusters are indicated with colored circles, and the related functions are reported below.

All proteins identified over the post-mortem time course were analyzed by the String software (https://version-11-0.string-db.org) for their involvement in biological mechanisms through the constitution of functional networks. By this analysis, it was possible to observe that the changes in the expression level of the proteins univocally identified for the e-PMI produced networks related to cytoskeletal remodeling (mediated by proteins connected to *Actb* as a hub protein), energetic metabolism (*Pgm1* and *2*) and cellular response to oxidative stress (*Sil1* and *Hyou1*). In addition, there was also a functional association of VMAC, Capn11, and DPA5A, which highlights the strong correlation between *Capn11* and the group of proteins involved in cytoskeletal remodeling and signal transduction. Furthermore, calpain 11 acts as a hub protein that connects the group of proteins involved in signaling transduction (*Capn2* and *Cast*) to *Psm4*, which plays a key role in cytoskeletal remodeling following protein degradation. Specifically, *Cast* inhibits CAPN11, which participates in post-mortem tenderization of meat and has been proposed to be involved in protein degradation muscle in living tissue [34] (Figure 5(a1,a2)).

As for the proteins identified for the l-PMI, the analysis of the pathways that are involved in the identified proteins includes three possible mechanisms: energy support by oxidative phosphorylation, mediated by the proteins of the respiratory chain (NDUFs), degradative pathways (MAP3K1) involved in the loss of normal cytomorphology and a regulatory process of cell death dependent on the caspase system and *Gsdma3* (Figure 5b).

Furthermore, *Ndfa9*, *Ripk1*, *Tirp53*, *Casp8,* and *Casp1* are the relevant hub proteins that allow to functionally associate the NDUFs with receptors of inflammation transduction and activators of programmed necrosis; regarding the latter, it is noteworthy the role of tumor protein 53 (related to the gene *Tirp53*) as a fundamental negative regulator of cell cycle by apoptosis via BAX and FAS mechanism [35]. Likewise, Casp8 and the other isoforms are included in the cluster responsible for programmed cell death. Finally, Casp1, involved in defense mechanisms against pathogens, is linked with Gsdma3, which promotes pyroptosis.

## 3. Discussion

The number of proteomics studies concerning brain samples has been increasing in recent years, but only a few of them are aimed at evaluating the association between proteomic changes and PMIs [16,19,36,37,38]. Since the number of protein markers currently used for forensic PMI estimation is still limited, the identification of additional protein markers would facilitate future application of this approach. Thus, the aim of this study was to profile post-mortem changes in the mouse cortex proteome to uncover further potential biomarkers useful for forensic purposes. Brain cortical samples have been used as it emerged from the literature that biochemical changes are more pronounced [39,40]. Neuroproteomic studies offer greater insights because neuronal proteins are closer to phenotype than transcripts. Moreover, the degradation of proteins after death is slower and more reproducible than the degradation of other biomarkers, such as RNAs [18].

In our experimental protocol, twenty-four 6-week-old female SAMR1 mice were sacrificed at T0. Apart from four of them (randomly chosen), the brain of which was immediately dissected and stored at −80 °C, thus representing the reference T0 sample, the remaining mice were stored at a constant temperature of 4 °C until brain dissection performed at different times after death. The choice of the storage temperature of animals is a determinant for brain proteomic studies. Although Hunsucker et al. [18], who analyzed the mouse brain tissue proteome, emphasized that during the post-mortem period, the large majority of proteins are stable up to 4 h when tissues are stored at room temperature (25 °C), degradation of proteins was more evident in samples maintained at room temperature for longer periods. Thus, we chose the temperature of 4 °C for animal storage based on the evidence that lower temperature down to 0 °C causes ice crystal formation and tissue damage; more importantly, in forensic practice, the cadavers are usually stored at that temperature for at least 24 h before being analyzed. The literature data underline the influence of this parameter on proteome changes. While the alterations of the skeletal muscle proteome are slower at 4 °C and, therefore, artifacts due to sample preservation are avoided [11,19], the same temperature can significantly affect the levels of many brain proteins [41]. These changes can occur in two different fashions, as discussed below, in that some proteins undergo massive proteolysis coupled with an increase in peptide fragments, whereas some other proteins tend to disappear along prolonged PMIs.

Indeed, the comparative analysis we performed among all gel images obtained at the different post-mortem time points highlighted an increasing trend of the total spot number detected over the PMI time course. This finding is in agreement with results from other research groups. For example, Di Luca et al. [42] investigated post-mortem proteome changes in porcine muscle exudate by 2-D DIGE and Western Blot analysis correlated the observed increase in the spot number over time to an accumulation of degradation products due to proteolytic enzyme activity. Also, Skold et al. [17] demonstrated that proteins are subjected to massive degradation after one minute post-mortem, generating several fragments. Therefore, the above-mentioned data, as well as some of ours, raise questions especially about the reliability of proteome maps related to more advanced post-mortem times, as they would not represent the real condition but the effect of massive proteolysis. For this reason, a greater correspondence to reality might be obtained in the early post-mortem stage (<24 h). However, this would require, for ethical reasons, the study of biomarkers obtained from a different matrix, such as saliva, blood, or urine, but no studies have been focused on these matrices for forensic pathology purposes. Furthermore, highly abundant proteins that could invalidate 2DE analysis characterize the above-mentioned matrices.

In relation to the identification of proteins reported in Table 1, there are several aspects that need to be emphasized. The first point is that MS, corroborated by LIFT technology and western blot analyses, allowed us to identify proteins with unfragmented structures. In particular, although cortical proteome showed a high grade of similarity both in the early and late post-mortem stages, gel comparison led to the identification of a number of unmatched spots, among which only those with a statistically significant intensity value (*p* < 0.05) and expression level ≥ 2 for subsequent identification in MS were selected. By these criteria, 22 unique proteins with a different expression and an intact molecular structure over the post-mortem time course (up to 0 32 h) were chosen (Table 1).

Among these 22 proteins, some were downregulated, while most of them were upregulated along different PMIs as compared to the same proteins at T0. Indeed, our analyses revealed that seven proteins were downregulated over the entire PMI (0–32 h, group a, Table 1). Interestingly, they were deputed to functions fundamental during life, so it is conceivable their post-mortem disappearance. For example, CTL3 is a protein deputed to transmembrane transporter activity in mice while showing a better affinity for choline transport in humans; PRS4 and PRS10 are also involved in protein turnover homeostasis. Of note is also the identification of AZI2. This protein exists in three ubiquitous different isoforms (Az1–Az3) capable of inhibiting the ODC pathway and polyamine uptake, exerting an important control of polyamine homeostasis. For this reason, AZI2 downregulation could be considered a PMI biomarker since, through polyamine homeostasis alteration, it promotes cell death/apoptosis [43]. Again, cellular depletion of PEA15 coupled to that of GFA and its filament disassembly has been reported in subpopulations of murine-injured astrocytes [44].

In contrast, both in e-PMI and l-PMI, there were a number of different proteins with upregulated expression in addition to the downregulated proteins discussed above. However, in the e-PMI, there was a peculiar behavior of the seven upregulated proteins, in that two of them showed an increased content only in the first 12 h post-mortem, while the other five proteins remained upregulated for a prolonged period, even up the l-PMI. The first two proteins are usually involved in carbohydrate metabolism, such as PGM2, and mitochondrial activity, like PAAT. This phenomenon has been reported in skeletal muscles in the early post-mortem phase [45], as well as in bone [46]. As for the other proteins with a persistent upregulation in e-PMI and l-PMI, they included SIL1, involved in protein folding in the ER and DPA5A, which could contribute to the activation of the protein synthesis machinery, possibly leading to an increase in the levels of two other structural cytoplasmic proteins such as VMAC and ACTB. These findings are compatible with anoxia occurring after death that can promote mechanisms aimed at recovering homeostasis, as it had emerged in response to cardiovascular injury, where mitochondrial dysfunction occurs and can lead to apoptosis and necrosis. In this context, mitochondria, together with endoplasmic reticulum (ER), are the major players in the response of cells to environmental perturbations [47]. Alternative explanations as to why protein synthesis may continue in the post-mortem period could be related to further post-mortem intracellular perturbations, including increased oxidative stress and cytosolic calcium levels. The former could, in turn, activate the synthesis of proteins related to the oxidative environment [47], while the latter could be responsible for the activation of Ca^2+^-dependent enzymes, as was the case of calpains. In agreement with this last observation, we found elevated levels of calpain-11 (CAN11_Capn11) starting from 12 h from death, which persisted in the l-PMI. Calpains are a 15-member family of Ca^2+^-activated cysteine proteases localized in the cytosol and mitochondria, and several of them have been shown to regulate apoptosis and necrosis [48]. Indeed, different stress factors in various cells (endothelial cells, renal cells, and cardiomyocytes) can promote activation of Calpain 1 and Calpain 2, which cause apoptosis and necrosis by cleaving cytoskeletal proteins, which in turn increase plasma membrane permeability and cleavage of caspases [3]. Additionally, Sanvicens et al. have shown that both caspases and calpains contribute to oxidative stress-induced apoptosis in retinal photoreceptor cells [49]. Thus, these findings suggest a remarkable role of calpain-11 as a putative biomarker of e-PMI and also l-PMI.

As for the proteins with an exclusively increased expression in the l-PMI, we found some proteins with heterogeneous functions like TCAL8, HS3S6, and NDUFAF7, while gasdermin-3A caught our attention as it plays an important role in membrane permeabilization and pyroptosis, that is a lytic pro-inflammatory type of cell death. Gasdermin 3A was detected in the late post-mortem stage in multiple isoforms with different pI as the result of post-translational changes (i.e., phosphorylation), whose role is still unknown. When gasdermin undergoes proteolytic cleavage, it releases two domains that, inserted into the cell membrane, forms wide pores that disrupt ion homeostasis and induce cell death [50]. Furthermore, gasdermin is also functionally linked to the caspase group through Caspase1. Of note, as confirmed by our data related to l-PMI (Figure 5b), caspases are involved in the apoptotic cascade. These findings reinforce the idea that gasdermin-3A might be considered a useful and selective marker for l-PMI estimation.

CASP7 and CASP8 also deserve particular consideration as possible selective l-PMI biomarkers as they started to be expressed from 24 to 32 h post-mortem. Caspases belong to a wide family of proteins that are evolutionarily highly conserved and are directly implicated in apoptosis. In particular, Caspase-8 is considered the principal initiator of the extrinsic apoptotic pathway, but under certain conditions, it can also trigger the intrinsic apoptotic pathway [51,52]. Furthermore, Kemp et al. [53] found that environmental stress, such as hypoxia, could cause an increasing mitochondrial permeabilization, which leads to the activation of Caspase 9.

Data from the bioinformatics classification via Gene Ontology analysis support the different roles of the proteins identified in the PMI and l-PMI stages. From a molecular function point of view, in the early stage, an equal distribution between binding proteins and proteins with catalytic activity was detected, while in the late stage, the latter was predominant. This finding confirms the hypothesis that in the first post-mortem phase (<24 h), the normal cellular biological processes related to biogenesis continue to take place, while in the late phase, the catalytic processes prevail due to the increase in protein degradation (Figure 4A). The distribution of biological processes shows evident differences in the two considered periods. Indeed, proteins involved in metabolic processes exhibit an important reduction in the late stage; in addition, after 24 h from death, two new categories were identified (cellular process and localization) (Figure 4B). This finding may be linked to the increase in protein degradation occurring in l-PMI.

With regard to cellular protein localization, in the early phase, there was a prevalence of cytoplasmic proteins, probably related to their release and consequent protein degradation processes. In the late stage, a new category of membrane proteins is noted. This finding is in contrast with literature data that depict a higher proteolytic activation with the disintegration of the membrane and release of the intracellular content in the late stages [3]. Furthermore, according to protein pathway analysis, only one category was identified in both early and late stages (Huntington’s disease). Of note is that the e-PMI pathways were mainly linked to neurodegenerative disorders, regulatory mechanisms of signal transduction, and inflammation. On the contrary, in the l-PMI, pathways linked to cellular death prevail (apoptosis and FAS signaling). This data is consistent with the analysis of protein interaction networks, which, in the early stage, underlines a constant interaction between the group of proteins that regulate cell proliferation mechanisms and the groups that regulate biogenesis and give genomic stability to the system by repairing DNA damage. This is also in agreement with the literature, where it has been reported that these events are frequent and similar to those occurring in early phases of pathological events associated with hypoxia/ischemia [52,53].

Finally, the analysis performed by using the STRING software corroborated the prominent role played by the proteins we have suggested as putative biomarkers in e-PMI and l-PMI. Indeed, in the e-PMI, calpain 11 could act as a hub protein able to control those involved in cytoskeletal remodeling following protein degradation, while in the l-PMI, the STRING analysis shows an evident functional interaction between the regulatory system of oxidative phosphorylation (NDUFs) and degradative pathways linked to the regulatory factors of cell death (caspase system). Altogether, the STRING analysis sheds light on the importance of two possible mechanisms that occur in the e-PMI and mainly in the l-PMI, not forgetting that most of the proteins upregulated in the e-PMI continue to be upregulated during l-PMI. The first one is a process of cell death dependent on the caspase system, and the second is a regulatory pathway concerning the cytoskeletal components capable of influencing the activation of the apoptosis process through an action that is carried out with cytoskeletal components changes, loss of normal cytomorphology as well as destabilization of the cytoskeleton.

## 4. Materials and Methods

### 4.1. Animals and Tissue Samples

Brain tissues were harvested from 6-week-old female SAMR1 mice (purchased from Charles River Laboratories Italia, Milan, Italy) under conditions that simulate the handling of human autopsy material. Twenty-four mice were sacrificed through cervical dislocation. In 4 mice randomly chosen, the cerebral cortex was immediately removed after death (T0) and treated as reported below, while the remaining 20 mice were stored at 4 °C soon after death to avoid artificial desiccation and contamination effects. Subsequently, the cerebral cortex was sampled from four randomly chosen animals at different PMIs, that is, at 4, 8, 12, 24, and 32 post-mortem hours.

The brain cortex was carefully dissected to avoid contamination by meninges and maintain intact tissue. All samples collected after each time period were rapidly washed in chilled PBS to remove blood, sectioned to pieces (of about 0.4 mg), that were placed in a vial containing 1 mL solution of protease inhibitors (Sigma-Aldrich, Milan, Italy), frozen in liquid nitrogen and stored at −80 °C until processing by further analyses.

All experiments were performed in accordance with the European Communities Council Directive of 22 September 2010 for the care of laboratory animals and after approval of the Local Ethics Committee of the University of Chieti-Pescara (PROG/48). All efforts were made to minimize the number of animals used.

### 4.2. Bidimensional Electrophoretic (2DE) Analysis

The brain cortex samples obtained from 24 mice were separately homogenized while the cytosolic proteins were extracted by lysis buffer, including urea (7 M), thiourea (2 M), CHAPS, tributyl-phosphine (2 mM), and a protease inhibitor cocktail mentioned above. Following protein concentration measurement by Better Bradford assay (Pierce), aliquots of 150 µg for analytical gels and 500 µg for preparative gels were rehydrated (DeStreak Rehydration Solution, GE Healthcare, Uppsala, Sweden) and analyzed by isoelectric focusing (IEF) (Ettan IPGphor III System, GE Healthcare, Uppsala, Sweden). The bidimensional electrophoresis (2DE) was performed according to procedures previously described by Angelucci et al. [54]. After gel staining and scanning (300 dpi), a reference gel was created, combining all spots at each time point, which was used to estimate the difference in protein expression.

After background subtraction, the intensity volume of each spot was normalized to the total intensity volume obtained by the sum of the intensity volumes of all spots identified in the same 2D gel. The intensity volumes of individual spots, calculated as mean ± SEM, were compared with those from different gels by one-way analysis of variance (ANOVA). Only protein spots with a probability (*p*) value < 0.05 were considered statistically significant and subjected to tryptic digestion and identification by mass spectrometry (MS).

### 4.3. Protein Digestion and MALDI TOF-TOF MS Analysis

Protein spots identified as reported above were excised from gels and analyzed by peptide mass fingerprinting (PMF) in a MALDI-TOF/TOF spectrometer. Each protein spot picked from gels after tryptic digestion was applied to a C18ZipTip (Millipore, Bedford, MA, USA) and eluted directly on the MALDI target by using 0.5 μL of saturated α-cyano-4-hydroxycinnamic acid (1:1, ACN:0.1% TFA) solution to be analyzed by Autoflex Speed mass spectrometer (Bruker Daltonics, Bremen, Germany) equipped with a Nd:YAG laser (355 nm, 1000 Hz) operated by FlexControl v3.3 and equipped with a 355-nm nitrogen laser. Detailed technique parameters have previously been reported [54,55]. In particular, the voltage parameters were set at IS1 19 kV, IS2 16.7 kV, lens 8.5 kV, reflector 1 21.0 kV, and reflector 2 9.7 kV. The delay time was 10 ns, and the acquisition mass-to-charge range was 500–4000 Th. A peptide mixture was used for external high precision calibration (HPC), containing bradykinin (fragment 1–7) 757.39 *m*/*z*, angiotensin II 1046.54 *m*/*z*, ACTH (fragment 18–39) 2465.19 *m*/*z*, Glu Fibrinopeptide B 1571.57 *m*/*z*, and renin substrate tetradecapeptide porcine 1760.02 *m*/*z*. Internal mass calibration was carried out using trypsin autodigestion products (843.50 *m*/*z*, 1046.56 *m*/*z*, 2212.11 *m*/*z*, 2284.19 *m*/*z*). PMF data obtained by MS were analyzed by databases (NCBI and Swiss Prot) through the Mascot search engine and comparing the experimental masses from tryptic digest with those theoretical of all selected protein spots, using as research parameters: PMF, trypsin, fixed modifications such as carbamidomethylation (Cys) as well as variable changes including methionine oxidation, monoisotopic mass, charge peptide state + 1, miss cleavage up to 1, and mass tolerance for each peptide at 100 ppm.

### 4.4. Protein Validation by LIFT Technology and Western Blot Analysis

All digested proteins produced a spectrum in PMF analysis with a range beyond *m*/*z* 700–3000 Da and were validated by LIFT MS/MS technology after selecting a maximum number of 4 precursor ions per sample to be subjected to MS/MS analysis. Analyses were performed in positive LIFT reflectron mode. The precursor Ion Selector (PCIS) range was 0.65% of the parent ion mass. For the database search, PMF and MS/MS data were combined using the BioTools 3.2 program connected to the Mascot search engine. The probability score, i.e., the matching between experimental data and peptide sequences deposited in the database with *p* < 0.05, was adopted as a criterion for correct identification. These scores were reported as log10 (P), P representing the maximum probability. The acceptable score values were 70 for PMF and 30/40 for MS/MS research.

As for western blot analysis, cortical tissue was minced and sonicated at 4 °C in a lysis buffer containing a protease inhibitor cocktail (Sigma-Aldrich). After centrifugation (14,000 rpm, 10 min, 4 °C) of the obtained cell suspension, the protein amount was measured by BioRad protein assay (Bio-Rad Laboratories, Milan, Italy). According to a standardized procedure previously published [56], protein samples (usually 50 μg), diluted in sodium dodecyl sulfate (SDS)-bromophenol blue buffer and boiled for 5 min, were run on 12% SDS polyacrylamide gels. Proteins were subsequently transferred on polyvinylidene fluoride (PVF) membrane, blocked with PBS/0.1% Tween20/5% nonfat milk (Bio-Rad Laboratories) for 2 h at 4 °C, and then incubated (overnight, 4 °C) with primary antibodies (polyclonal rabbit anti-Calpain 11, dilution 1:500, from Invitrogen by Thermo-Fisher Scientific, Italy; monoclonal rabbit anti-Gasdermin-3, dilution 1:200, and polyclonal mouse anti-Caspase 8, dilution 1:1000, both purchased from Sigma-Aldrich, Milano, Italy), followed by a second incubation (1 h, room temperature) with goat anti-rabbit HPR-conjugated secondary antibody (final dilution 1:5000, Bethyl Laboratories Inc.; Montgomery, TX, USA). Finally, immunocomplexes were visualized by chemiluminescence (ECL) detection system (GE Healthcare Life Sciences, Milan, Italy) and quantified by densitometric analysis (ImageJ software, https://imagej.net/ij/nih-image; U.S. National Institutes of Health, Bethesda, MD, USA).

### 4.5. Bioinformatic Analysis

Data obtained by the MS analysis were imported in the Protein Analysis THrough Evolutionary Relationship (PANTHER) (http://www.pantherdb.org/, SRI International, Menlo Park, CA, USA) and Gene Ontology (GO) database to elicit the molecular function, biological process, and cellular distribution of the proteins unequivocally expressed at early and late PMI stage. Using then the software STRING (http://string-db.org), it was also possible to build networks between the protein identified in the mouse cortex at different PMIs and other proteins based on known direct and indirect interactions described in the literature. A confidence level of 95% was considered the cut-off for the analysis.

### 4.6. Data Analysis

All experiments were performed in at least two independent biological replicates and analyzed for statistical significance as indicated. Whenever applicable, numerical values are reported as mean ± E.S., being differences considered as statistically significant at *p* < 0.05 (*t* Student, one way).

## 5. Conclusions

In conclusion, the ability to assess the impact of post-mortem time-dependent changes on the proteome is critical to reveal death-associated changes in protein expression. The focus of the present study was not to produce a complete list including all possible alterations in detail but rather to identify a number of possible markers to be used as sensitive, reliable indicators of proteolytic activity that are changed as a function of post-mortem time. To date, none of the proteins mentioned have been identified in previous investigations. Moreover, all proteins identified are non-tissue specific and, therefore, could be detectable in other organs, proving useful as PMI biomarkers even if the proteomic analysis was performed using different body tissues.

Although we are aware that further studies need to confirm the validity of our results, they suggest that e-PMI proteome changes are more representative of the real protein alterations rather than l-PMI changes. Nevertheless, the results obtained by the functional interaction analysis suggest that l-PMI could be evaluated through caspase family monitoring, although further investigations should focus on quantitative differences of all caspase isoforms at each post-mortem time.

## Figures and Tables

**Table 1:** Changes in the expression of mouse cortex proteins evaluated at different PMIs.

**(a) *Top proteins downregulated in mouse cortex from 0 to 32 h***									
**SPOT** **ID**	**Abbreviated Name_Gene Name**	**AC ^a^ Swiss**	**Protein Description**	**Score ^b^**	**Peptide** **Matched**	**SC ^c^ %**	**Theoretical** **(pI/Mr)**	***p*-Value**	**Variation**
EXP1	PRS4_*Psmc1*	P62192	26S proteasome regulatory subunit 4	41	6/38	49	5.87/59184	0.0076	DOWN
EXP2	CTL3_*Slc44a3*	Q921V7	Choline transporter-like protein 3	44	5/18	57	8.04/73414	0.0112	DOWN
EXP4	PRS10_*Psmc6*	P62334	26S proteasome regulatory subunit 10B	67	21/33	78	7.09/44172	0.0008	DOWN
EXP5	AZI2_*Azi2*	Q9QYP6	5-azacytidine-induced protein 2	44	11/59	65	6.23/46062	0.0113	DOWN
EXP6	PDCL2_*Pdcl2*	Q78Y63	Phosducin-like protein 2	51	6/21	39	5.00/22134	0.0021	DOWN
EXP7	PEA15_*Pea15*	Q62048	Astrocytic phosphoprotein PEA-15	60	5/56	46	4.94/15054	0.0300	DOWN
EXP8	GABPB-1_*Gabpb1*	Q00420	GA-binding protein subunit beta-1	64	6/25	39	4.76/41331	0.0011	DOWN
**(b) *Top proteins induced in mouse cortex at early PMI (from 0 to 24 h)***									
**SPOT** **ID**	**Abbreviated Name_Gene Name**	**AC ^a^ Swiss**	**Protein Description**	**Score ^b^**	**Peptide** **Matched**	**SC ^c^ %**	**Theoretical** **(pI/Mr)**	***p*-Value**	**Variation**
ER1	PGM2_*Pgm2*	Q7TSV4	Phosphoglucomutase-2	57	19/46	36	5.78/69274	0.0018	UP
ER2	PAAT_*Paat*	Q9D2Q3	ATPase PAAT Protein associated with ABC transporters	60	22/95	55	6.03/48743	0.0400	UP
T12_1	VMAC_*Vmac*	Q8BP01	Vimentin-type intermediate filament-associated coiled-coil protein	30	5/23	30	5.21/9063	0.0021	UP
T12_2	DPA5A_*Dppa5a*	Q9CQS7	Developmental pluripotency-associated protein 5A	30	8/85	46	6.15/13858	0.0052	UP
T12_7	SIL1_*Sil1*	Q9EPK6	Nucleotide exchange factor SIL1 precursor	57	15/58	47	5.20/52429	0.0030	UP
T12_8	CAN11_*Capn11*	Q6J756	Calpain-11	50	11/24	52	6.03/82969	0.0213	UP
T12_9	ACTB_*Actb*	P60710	Actin, cytoplasmic 1	51	20/99	48	5.29/42052	0.0051	UP
**(c) *Top proteins induced in mouse cortex at late PMI (from 24 to 32 h)***									
**SPOT** **ID**	**Abbreviated Name_Gene Name**	**AC ^a^ Swiss**	**Protein Description**	**Score ^b^**	**Peptide** **Matched**	**SC ^c^ %**	**Theoretical** **(pI/Mr)**	***p*-Value**	**Variation**
A6_3	NDUFAF7_*Ndufaf7*	Q9CWG8	Protein arginine methyltransferase, mitochondrial precursor	70	21/53	48	6.47/48384	0.0090	UP
A6_4	NPM_*Npm1*	Q61937	Nucleophosmin	45	8/25	53	4.62/32560	0.0118	UP
A6_6	GSDA3_*Gsdma3*	Q5Y4Y6	Gasdermin-A3	62	12/46	59	5.53/51987	0.0015	UP
A7_2	CASP8_*Casp8*	O89110	Caspase-8	52	28/122	51	5.12/56291	0.0077	UP
A7_6	HS3S6_*Hs3st6*	Q5GFD5	Heparan sulfate glucosamine 3-O-sulfotransferase 6	58	8/28	42	10.55/37415	0.0104	UP
T24_1	CASP7_*Casp7*	P97864	Caspase-7	35	15/106	55	5.93/34666	0.0023	UP
T24_4	GSDA3_*Gsdma3*	Q5Y4Y6	Gasdermin-A3	59	10/57	44	5.53/51987	0.0103	UP
T24_5	TCAL8_*Tceal8*	Q9CZY2	Transcription elongation factor A protein-like 8	36	6/62	50	5.41/13578	0.0270	UP

All the identified proteins relate to *Mus musculus. *^a^ AC is the accession number in the Swiss-Prot database. ^b^ Score is −10 Log(P), where P is the probability that the observed match is a random event; it is based on the Swiss-Prot database using the MASCOT search engine. ^c^ Sequence coverage means the ratio of portion sequence covered by matched peptide to the full length of the protein sequence.

**Table 2:** Validation of the protein sequences of some proteins by LIFT Technology.

Label	Abbr. Name	Mw/pI Theor.	PMF Score ^a^	Peptide Matched/ Peptide Searched	SC ^b^ %	Lift (MS_2_) Ion Parent Masses (*m/z*)	Score ^c^ Tof-Tof	Peptide Sequence
T12_8	CAN11	6.03/82.96	50	11/24	52	1740.845 1453.768 1139.587	150	YRDHGFSEIFINSR DADFLLRVFTEK TKGFSLEVCR
A6_6	GASDA3	5.53/51.98	62	12/46	59	2122.195 2455.380 1390.787	230	HNLCALYAGLSLLHLLSRK ILPVQLKLVESTLEQNFLQD AVTIPKGCVLAYR
A7_6	CASP8	5.12/5629	52	28/122	51	2019.022 1101.601 909.427	187	NKPRGYCLIINNHDFSK AREDITQLR SESRTSDK

^a^ PMF Score: values are Log_10_ (P), where P is the probability that the observed match is a random event, as inferred by the Swiss Prot database using the MASCOT searching program; ^b^ SC: Sequence Coverage means the ratio between the portion of the sequence covered by matched peptide and the full length of the protein sequence. ^c^ Score Tof-Tof: a score that results from combining PMF and MS/MS matched peptides from ion parent fragments.

## Data Availability

Data supporting the results are shown in the figures. Row data can be made available upon reasonable request to the corresponding author.

## Supplementary Materials

The following supporting information can be downloaded at: https://www.mdpi.com/article/10.3390/ijms25168736/s1.
