# Supplementary figures and images for: Proteomic Characterization of Changes in Mouse Brain Cortex Protein Expression at Different Post-Mortem Intervals: A Preliminary Study for Forensic Biomarker Identification
Source: Int J Mol Sci. 2024 Aug 10;25(16):8736. doi: 10.3390/ijms25168736 (PMC11354345; doi:10.3390/ijms25168736)

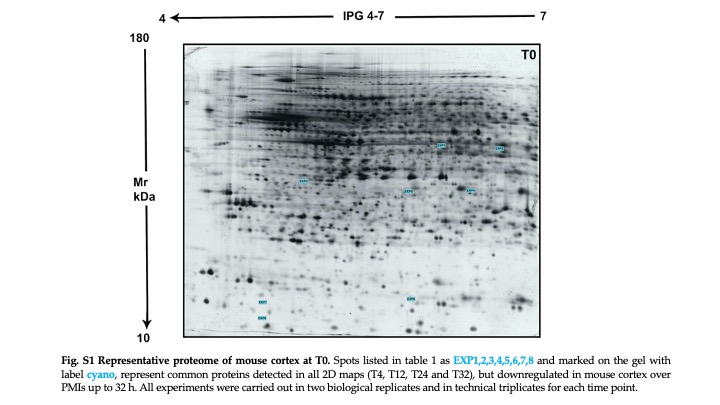

Supplement: Supplementary file 1 [file ijms-25-08736-s001.zip › Figure S1.jpg]

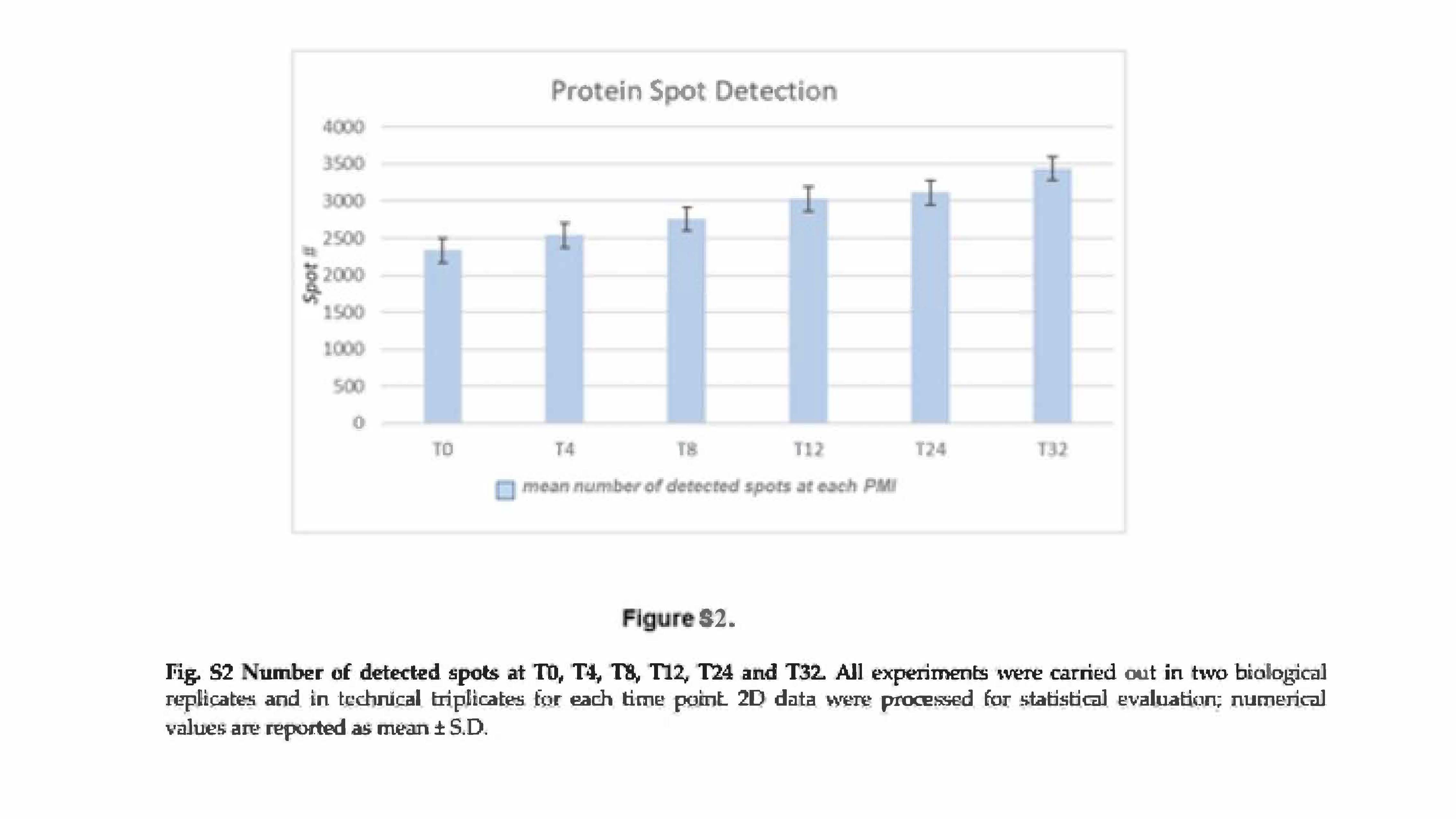

Supplement: Supplementary file 1 [file ijms-25-08736-s001.zip › Figure S2.jpg]
